# Ryanodine receptor-mediated Ca^2**+**^ release underlies iron-induced mitochondrial fission and stimulates mitochondrial Ca^2**+**^ uptake in primary hippocampal neurons

**DOI:** 10.3389/fnmol.2014.00013

**Published:** 2014-03-11

**Authors:** Carol D. SanMartín, Andrea C. Paula-Lima, Alejandra García, Pablo Barattini, Steffen Hartel, Marco T. Núñez, Cecilia Hidalgo

**Affiliations:** ^1^Center for Molecular Studies of the Cell, Institute of Biomedical Sciences, Faculty of Medicine, Universidad de ChileSantiago, Chile; ^2^Biomedical Neuroscience Institute, Faculty of Medicine, Universidad de ChileSantiago, Chile; ^3^Institute for Research in Dental Sciences, Faculty of Dentistry, Universidad de ChileSantiago, Chile; ^4^Laboratory of Scientific Image Processing, Anatomy and Developmental Biology Program, Institute of Biomedical Sciences, Faculty of Medicine, Universidad de ChileSantiago, Chile; ^5^Department of Biology, Faculty of Sciences and Research Ring on Oxidative Stress in the Nervous System, Universidad de ChileSantiago, Chile; ^6^Physiology and Biophysics Program, Institute of Biomedical Sciences, Faculty of Medicine, Universidad de ChileSantiago, Chile

**Keywords:** endoplasmic reticulum, reactive oxygen species, mitochondrial calcium, cellular redox state, mitochondrial network, Drp-1

## Abstract

Mounting evidence indicates that iron accumulation impairs brain function. We have reported previously that addition of sub-lethal concentrations of iron to primary hippocampal neurons produces Ca^2^^+^ signals and promotes cytoplasmic generation of reactive oxygen species. These Ca^2^^+^ signals, which emerge within seconds after iron addition, arise mostly from Ca^2^^+^ release through the redox-sensitive ryanodine receptor (RyR) channels present in the endoplasmic reticulum. We have reported also that addition of synaptotoxic amyloid-β oligomers to primary hippocampal neurons stimulates RyR-mediated Ca^2^^+^ release, generating long-lasting Ca^2^^+^ signals that activate Ca^2^^+^-sensitive cellular effectors and promote the disruption of the mitochondrial network. Here, we describe that 24 h incubation of primary hippocampal neurons with iron enhanced agonist-induced RyR-mediated Ca^2^^+^ release and promoted mitochondrial network fragmentation in 43% of neurons, a response significantly prevented by RyR inhibition and by the antioxidant agent *N*-acetyl-L-cysteine. Stimulation of RyR-mediated Ca^2^^+^ release by a RyR agonist promoted mitochondrial Ca^2^^+^ uptake in control neurons and in iron-treated neurons that displayed non-fragmented mitochondria, but not in neurons with fragmented mitochondria. Yet, the global cytoplasmic Ca^2^^+^ increase induced by the Ca^2^^+^ ionophore ionomycin prompted significant mitochondrial Ca^2^^+^ uptake in neurons with fragmented mitochondria, indicating that fragmentation did not prevent mitochondrial Ca^2^^+^ uptake but presumably decreased the functional coupling between RyR-mediated Ca^2^^+^ release and the mitochondrial Ca^2^^+^ uniporter. Taken together, our results indicate that stimulation of redox-sensitive RyR-mediated Ca^2^^+^ release by iron causes significant neuronal mitochondrial fragmentation, which presumably contributes to the impairment of neuronal function produced by iron accumulation.

## INTRODUCTION

Under physiological conditions, mitochondria undergo constant structural changes, forming interconnected networks or punctiform organelles depending on cell type (Kuznetsov et al., 2009). Cells finely regulate changes in mitochondrial network structure, maintaining a continuous balance between mitochondrial fission and fusion (Detmer and Chan, 2007; Knott et al., 2008). In mammals, the hFis1 protein and the large GTPase Dynamin-related protein 1 (Drp1) are key molecules for the regulation of mitochondrial fission (Smirnova et al., 2001). The hFis1 protein is anchored to the outer mitochondrial while the Drp1 protein is predominantly localized in the cytoplasm.

Mitochondria acting as Ca^2^^+^ buffers participate in intracellular Ca^2^^+^ homeostasis. Due to the low Ca^2^^+^ affinity of the mitochondrial Ca^2^^+^ uniporter, effective mitochondrial Ca^2^^+^ uptake requires the proximity of mitochondria to endoplasmic reticulum (ER) or plasma membrane Ca^2^^+^ channels, since their opening generates transient microdomains of high Ca^2^^+^ concentration, a requisite feature of mitochondrial Ca^2^^+^ uptake (Spat et al., 2008). Neurons in primary culture (Zampese et al., 2011) and neuronal cells lines (Spat et al., 2008) exhibit a physical and functional association between the ER and mitochondria. This association allows mitochondrial Ca^2^^+^ uptake through the uniporter following Ca^2^^+^ release mediated by the ER resident Ca^2^^+^ channels, the ryanodine receptor (RyR; Szalai et al., 2000) and the inositol 1,4,5-trisphosphate (IP_3_) recepto (Csordas et al., 2006).

Calcium signals influence mitochondrial dynamics. Previous reports have described that Ca^2^^+^ influx promotes mitochondrial fission through activation of different Ca^2^^+^-sensitive downstream effectors, including the protein kinases PKA (Cribbs and Strack, 2007) and CaMKIα (Han et al., 2008) and the protein phosphatase calcineurin (Cereghetti et al., 2008). These effectors activate Drp1 by different mechanisms and promote its translocation to the mitochondria, where Drp1 interacts with hFis1 forming large complexes at future scission sites (cut sites) promoting mitochondrial fission (Frank et al., 2001). Additionally, we have reported that activation of RyR-mediated Ca^2^^+^ release with a selective RyR agonist-induces mitochondrial fragmentation in primary hippocampal neurons (SanMartin et al., 2012).

An imbalance in mitochondrial dynamics affects mitochondrial localization, changing their interactions with the ER, mitochondrial Ca^2^^+^ uptake and intracellular Ca^2^^+^ homeostasis (Pizzo and Pozzan, 2007). The propagation of mitochondrial Ca^2^^+^ waves in fragmented mitochondria is two times slower than in interconnected mitochondria (Frieden et al., 2004) while over-expression of the Drp-1 protein, which enhances fission, reduces the mitochondrial Ca^2^^+^ signals induced by RyR-mediated Ca^2^^+^ release in skeletal myotubes (Eisner et al., 2010). Jointly, these results confirm the importance of the mitochondrial fusion/fission balance to maintain Ca^2^^+^ homeostasis and indicate that an increase in mitochondrial fission decreases mitochondrial Ca^2^^+^ uptake (Eisner et al., 2013).

Oxidative stressors and some neurotoxic agents increase mitochondrial fission *in vitro *(Rintoul et al., 2003; Pletjushkina et al., 2006; Han et al., 2008). Moreover, cerebellar granule neurons treated with hydrogen peroxide exhibit mitochondrial fission as a previous event to apoptotic cell death; DNA damage induced by an inhibitor of topoisomerase produces similar effects (Jahani-Asl et al., 2007). In cortical neurons, reactive nitrogen species such as nitric oxide increase the number of mitochondria but decrease their size, promote Bax translocation to the mitochondria and diminish cellular ATP levels (Barsoum et al., 2006; Yuan et al., 2007). Accordingly, a defect in mitochondrial dynamics – such as persistent mitochondrial fission – might be one of the causes of the mitochondrial dysfunctions reported in some neurodegenerative diseases characterized by an increased cellular oxidative tone.

Increased basal Ca^2^^+^ levels and abnormal Ca^2^^+^ signaling, increased production of reactive oxygen species (ROS) and iron accumulation are characteristic hallmarks of Alzheimer’s disease, a highly prevalent neurodegenerative disease (Orth and Schapira, 2001). The accumulation and aggregation of amyloid-β (Aβ) peptide in the brain presumably causes the memory loss exhibited by individuals affected with Alzheimer’s disease (Paula-Lima et al., 2013). The brain of Alzheimer’s disease subjects contains two types of toxic Aβ aggregates, insoluble Aβ fibrils and soluble diffusible Aβ oligomers, both of which induce mitochondrial fission (Wang et al., 2009; Paula-Lima et al., 2011). We have shown previously that RyR channel inhibition prevents the mitochondrial network fragmentation produced by addition of sub-lethal concentrations of Aβ oligomers in primary hippocampal neurons (Paula-Lima et al., 2011), suggesting that RyR-mediated Ca^2^^+^ release plays an important role in the increased mitochondrial fission observed in Alzheimer’s disease.

A positive connection between neuronal iron levels and Ca^2^^+^ signaling has been uncovered recently. This association is based on finding that iron-induced ROS generation promotes RyR-mediated Ca^2^^+^ release in cultured neurons (Munoz et al., 2006, 2011; Hidalgo et al., 2007; Hidalgo and Nunez, 2007). Iron-induced stimulation of RyR-mediated Ca^2^^+^ release is likely to arise from RyR oxidative modifications induced by iron-generated ROS, since hydrogen peroxide addition to primary hippocampal neurons increases oxidative modifications of RyR cysteine residues and promotes RyR-mediated Ca^2^^+^ release (Kemmerling et al., 2007). We reported also (Munoz et al., 2011) that the increase in cytoplasmic free Ca^2^^+^ concentration due to iron-induced RyR stimulation enhances phosphorylation and nuclear translocation of the extracellular-signal-regulated kinases (ERK1/2), a requisite step of long-lasting synaptic plasticity (Thomas and Huganir, 2004).

Although normal neuronal function requires an optimal oxidative tone (Thannickal and Fanburg, 2000; Kennedy et al., 2012), excessive ROS levels are especially deleterious. A large amount of evidence describes the association between oxidative damage generated by increased ROS and the pathogenesis of neurodegenerative diseases such as Parkinson’s disease and Alzheimer’s disease (Marchesi, 2011; Schrag et al., 2013; Yan et al., 2013). Iron accumulation is another common feature not only of Alzheimer’s disease but also of other neurodegenerative disorders, including Parkinson’s disease (Zecca et al., 2004; Smith et al., 2010; Nunez et al., 2012). The causes underlying iron dyshomeostasis are unknown, and may have particular components in specific diseases, but mitochondrial dysfunction, inflammatory stimuli, decreased glutathione content, and oxidative damage seem to engage jointly in a positive feedback loop that results first in loss of neuronal function and then in neuronal death (Nunez et al., 2012). There is a direct relationship between iron accumulation and increased ROS levels, because iron participates in a group of redox reactions known collectively as the “Haber–Weiss/Fenton reactions,” which generate superoxide anion, hydrogen peroxide, and hydroxyl radicals. This set of reactions respond to mass-action law and have a large negative free energy change; thus, a direct relationship between the concentration of redox-active iron and ROS production can be established (Kehrer, 2000; Nunez et al., 2012).

Here, we investigated in primary hippocampal neurons the effects of iron treatment on mitochondrial network dynamics and function. We found that 24 h exposure of primary hippocampal cultures to iron promoted significant mitochondrial network fragmentation in a sizable fraction of neurons; this fragmentation process required functional RyR channels and was prevented by *N*-acetyl-L-cysteine (NAC). We also show that RyR-mediated Ca^2^^+^ release stimulated mitochondrial Ca^2^^+^ uptake only in iron-treated neurons that displayed a non-fragmented mitochondrial network structure.

## MATERIALS AND METHODS

### MATERIALS

Fluo4-AM, calcein-AM, MitoTracker Orange CMTMRos and Alexa Fluor^®^488 anti-mouse were from Molecular Probes (Carlsbad, CA, USA). Neurobasal medium, B27 supplement, and horse serum were from Gibco (Carlsbad, CA, USA). Dulbeco’s modified essential medium (DMEM) and lipofectamine 2000 were from Invitrogen (Carlsbad, CA, USA). Ryanodine was from Alexis (Lausen, Switzerland). The mito-pericam plasmid was a kind gift from Dr. Eisner. Protease inhibitors leupeptin and pepstatin A were from Calbiochem (La Jolla, CA, USA), and 3-[4,5-dimethylthiazol-2-yl]-2,5-diphenyl tetrazolium bromide (MTT) was from Sigma Chemical (St Louis, MO, USA). Bicinchoninic acid assay kit, horseradish peroxidase (HRP)-conjugated secondary antibodies and m-Hsp-70 antibody were from Pierce Biotecnology (Rockford, IL, USA), 4-cloro-methyl-cresol was from Merck (Darmstadt, Germany) and polyvinylidene difluoride (PDVF) membranes from Millipore (Bedford, MA, USA). The antibody anti-Cox IV was from Cell Signaling (Denvers, MA, USA), anti-Drp-1 was from Thermo Scientific (Salt Lake City, UT, USA), anti m-Hsp-70 was from Pierce Biotecnology (Rockford, IL, USA).

### PRIMARY RAT HIPPOCAMPAL CULTURES

Cultures were prepared from 18 day old embryos obtained from pregnant Sprague–Dawley rats as previously described (Paula-Lima et al., 2005). Briefly, after removal brains were placed in a dish containing Hank’s-glucose solution. Hippocampi were dissected and, after stripping away meninge membranes, cells were mechanically dissociated gently in Hank’s-glucose solution, centrifuged and resuspended in DMEM supplemented with 10% horse serum. Dissociated hippocampal neurons were plated on polylysine-treated plates. After 40 min, DMEM was replaced by Neurobasal medium supplemented with B-27. Cells were incubated for 18–21 days *in vitro* (DIV) at 37^o^C in a humidified 5% CO_2_ atmosphere prior to experimental manipulations. The resulting cultures were highly enriched in neuronal cells (Paula-Lima et al., 2011), with a glial content <24%.

### SUBCELLULAR FRACTIONATION

Mitochondrial fractions were obtained by differential centrifugation of hippocampal homogenates as previously described (Parra et al., 2008). Cells were scraped and lysed in ice-cold buffer A (in mM: 250 sucrose, 10 KCl, 1.5 MgCl_2_, 1 EDTA, 1 EGTA, 1 DTT, 20 Hepes/Na^+^, pH 7.4, plus protease inhibitors: 1 μg/ml leupeptin and 8 μg/ml pepstatin A). Cells were homogenized using a homogenizer with a tight fitting Teflon pestle. The homogenates were maintained on ice for 5 min and then centrifuged (500 × *g*, 10 min) to remove nuclei and unbroken cells. The supernatants were centrifuged (10,000 × *g*, 25 min) to obtain a pellet enriched in mitochondria, which was re-suspended in buffer A supplemented with 1% Triton X-100 and centrifuged (10,000 *g*, 25 min). The protein content in the resultant purified pellet, highly enriched with mitochondria, was determined by using the Bicinchoninic acid assay.

### IMMUNOCYTOCHEMISTRY

Hippocampal cultures were fixed by adding equal volumes of 4% formaldehyde and 4% sucrose fixation solution, in phosphate buffered saline (PBS) to the culture medium for 5 min. The fixation solution was replaced by fresh solution in the absence of culture medium, and incubation was continued for 10 min. Cells were rinsed three times with PBS and were incubated with 0.1% Triton X-100 in PBS (permeabilization solution) for 30 min and blocked with 5% BSA in PBS (blocking solution) for 1 h. Cells were immunostained with anti-Hsp-70 primary antibody (1:750, diluted in blocking solution) at 4°C overnight. After this incubation period, cells were rinsed three times with PBS and were incubated with Alexa Fluor^®^ 488 anti-mouse secondary antibody (1:400, diluted in blocking solution) for 1 h at room temperature. Cells were rinsed three times with PBS, and the coverslips were mounted in DAKO mounting medium on glass slides. To control for the specificity of Hsp-70 as a mitochondrial label, cultures were stained with 50 nM MitoTracker Orange for 20 min at 37°C.

### MORPHO-TOPOLOGICAL ANALYSIS

*(a) Fluorescence:* Confocal image stacks were captured with a Zeiss LSM-5, Pascal 5 Axiovert 200 microscope, using LSM 5 3.2 image capture, analysis software and a Plan-Apochromat 40x, 1.4 oil differential interference contrast objective. One-channel Alexa Fluor 488 (λ_ex_/λ_em_ = 488/505–530 nm) and image stacks [intensity I(x,y,z), voxel size Δx/Δy/Δz = 50/50/300 nm] were acquired. We made sure that I(x,y,z) did not saturate and that image background was slightly above zero by adjusting the laser power, the detector gain, and the detector offset. Image stacks were deconvoluted with Huygens Scripting (Scientific Volume Imaging, Hilversum, Netherlands). All image-processing routines were developed in our laboratory based on IDL (Interactive Data Language, ITT, Boulder, CO, USA). Mitochondria were identified by staining fixed cultures with Hsp-70, as described in our earlier work (Paula-Lima et al., 2011; SanMartin et al., 2012). The specificity of Hsp-70 as a mitochondrial stain was confirmed by staining mitochondria with MitoTracker Orange, which yielded the same labeling pattern as Hsp-70 (see Results). To determine mitochondrial protein Hsp-70 in neurites and soma, segmentations were performed to define different regions of interests (ROIs). First, the cross-section of neurons was segmented by an intensity threshold in the green fluorescence channel. Remaining holes inside the cells and artifacts outside the cells were filled or removed by morphological filters. For all experiments, the protocols remained constant, and the quality of the segmentation was controlled interactively by overlaying the original fluorescent images with the segmented ROIs. (*b)* *Determination of mitochondrial protein Hsp-70 in soma and neurite volumes by 3D reconstruction of the segmented objects*: 3D models were reconstructed from successive xy-images along the z-axis. Based on their volumes, we defined four different clusters to characterize mitochondrial connectivity. First, we determined the mean volume of single mitochondria, yielding 0.15 ± 0.04 μm^3^ (mean ± SE, *N* = 834). The mean volume of single mitochondria was used to define connected clusters: (i) 1–3 mitochondria (0–0.45 μm^3^, red objects; (ii) 4–10 mitochondria (0.45–1.5 μm^3^, green objects; (iii) 11–50 mitochondria (1.5–7.5 μm^3^, blue objects); (iv) over 50 mitochondria (>7.5 μm^3^, yellow objects).

### QUANTIFICATION OF FRAGMENTED MITOCHONDRIA

Image deconvolution was performed with the Image J software and z-stacks from images were projected in one image, which was analyzed. In accordance to mitochondrial morphology, neurons were classified as exhibiting fragmented or continuous mitochondrial network, by visual examination in ten optical fields for each condition, counting 15 neurons approximately in each field. The percentage of cells with a fragmented pattern was determined respect to the total number of cells analyzed.

### WESTERN BLOT ANALYSIS

Fractions enriched in mitochondrial proteins were resolved by 12% SDS-PAGE and then transferred to PDVF membranes. Blots were blocked for 1 h at room temperature in Tris-buffered saline (TBS) containing 0.2% Tween-20 and 5% fat-free milk. Overnight incubation with primary antibody against Drp-1 (1:1000) was performed at 4°C. After incubation for 1.5 h with HRP-conjugated secondary antibodies, membranes were developed by enhanced chemiluminescence (Amersham Biosciences, Bath, UK). To correct for loading, membranes were stripped and blotted against Cox-IV (1:1000) or mHsp-70 (1:1000). The films were scanned and the Image J program was employed for densitometric analysis of the bands.

### DETERMINATION OF CYTOPLASMIC Ca^2**+**^ SIGNALS

Cultures treated for 24 h with 30 μM Fe-NTA [FeCl_3_- sodium nitrilotriacetate complex (2.2:1 molar ratio)] or vehicle in Neurobasal medium supplemented with B-27 were placed in modified Tyrode solution (in mM: 129 NaCl, 5 KCl, 2 CaCl_2_, 1 MgCl_2_, 30 glucose, 25 HEPES/Tris, pH 7.3) prior to fluorescence measurements. This medium change was performed in order to avoid color interference from the Neurobasal medium. Cultures were loaded next with 5 μM Fluo4-AM for 30 min at 37°C. After washing three times with modified Tyrode solution, cultures were maintained in this solution during the experiment. Neurons were stimulated with 4-cloro-methyl-cresol (4-CMC) at the microscope stage (which was not removed from the medium). Fluorescence images of intracellular Ca^2^^+^ signals in primary hippocampal neurons were recorded every 5 s in a Carl Zeiss LSM Pascal 5 confocal microscope system using 63x Oil DIC objective, excitation 488 nm, argon laser beam. Frame scans were averaged using the equipment data acquisition program. Ca^2^^+^ signals are presented as F/F_o_ values, where F corresponds to the experimental fluorescence and F_o_ to the basal fluorescence.

### DETERMINATION OF MITOCHONDRIAL Ca^2**+**^ SIGNALS

Cultures grown in 25 mm glass plates were transiently transfected with the mito-pericam plasmid at 14 or 15 DIV using a proportion of 1:3 DNA:lipofectamine 2000. 1 day post-transfection, cultures incubated in Neurobasal medium supplemented with B-27 were treated with 30 μM Fe-NTA for 24 h, rinsed three times with modified Tyrode solution, and were maintained in this solution during the experiment. At the microscope stage, cultures were stimulated with 4-CMC (which was not removed from the medium) and mitochondrial Ca^2^^+^ signals from neuronal cells were recorded every 3 s in an Olympus Disk Scanning Unit (DSU) confocal microscope (Olympus, Hamburg, Germany). We identified neuronal cells by morphology: neuronal cells are smaller than glial cells and have a well-defined soma, with a volume larger than of the flatter glial cells. Changes in mitochondrial Ca^2^^+^ levels are presented as F/F_o_ values, where F corresponds to the experimental fluorescence and F_o_ to the basal fluorescence.

### STATISTICS

Results are expressed as mean ± SEM. All data (with *n* ≥ 9) complied with the normality distribution, determined by the Shapiro–Wilk test. Unless indicated otherwise, the significance of differences was evaluated using Student’s *t*-test for paired data, and with one-way ANOVA or two-way ANOVA followed by Bonferroni’s *post hoc* test for multiple determinations.

## RESULTS

### IRON ENHANCES AGONIST-INDUCED RyR-MEDIATED Ca^2**+**^ RELEASE IN HIPPOCAMPAL NEURONS

**FIGURE 1:**
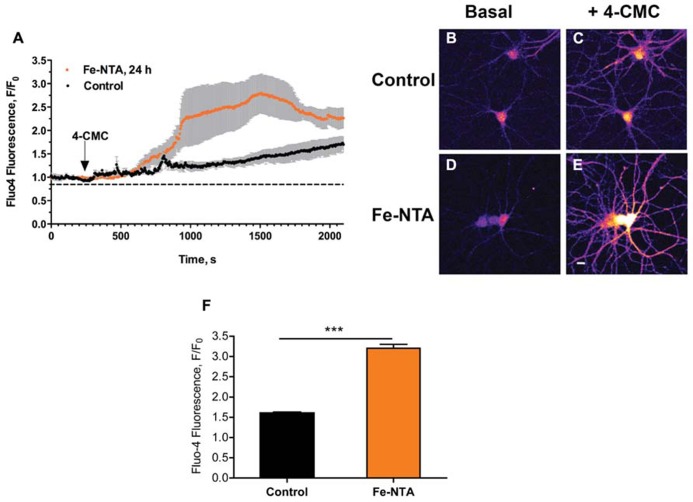
**Iron treatment increases agonist-induced RyR-mediated Ca^2^^+^ release in primary hippocampal neurons**. **(A)** Representative time course of Fluo4 fluorescence changes recorded before and after addition of 0.5 mM 4-CMC to control neurons (black symbols) or to neurons treated with 30 mM Fe-NTA for 24 h (orange symbols). Pseudo color images of confocal sections obtained from control neurons loaded with Fluo4-AM recorded before **(B)** and after **(C)** addition of 0.5 mM 4-CMC, or from neurons treated with Fe-NTA for 24 h taken before **(D)** and after **(E)** addition of 0.5 mM 4-CMC. In **(C)** and **(E)**, images correspond to the maximal fluorescence increase. Calibration bar = 20 μm. **(F)** Maximum fluorescence increase, recorded in the neuronal soma, elicited by addition of 0.5 mM 4-CMC to control cultures or to iron-treated cultures. Values represent Mean ± SE (*n* = 4–5 independent cultures, 4–7 cells analyzed per culture). Statistical significance was analyzed by Student’s paired *t*-test; ***p < 0.0001.

We have shown previously that iron addition to primary hippocampal neurons increases intracellular ROS levels, and generates cytoplasmic Ca^2^^+^ signals within seconds through stimulation of RyR-mediated Ca^+^^2^ release from the ER (Munoz et al., 2011). Here, we evaluated if prolonged exposure (24 h) of cultured hippocampal neurons to 30 μM Fe-NTA affected agonist-induced RyR-mediated Ca^2^^+^ release. To this aim, cultures treated with Fe-NTA were loaded with the Ca^2^^+^ indicator Fluo4-AM and stimulated with the RyR channel agonist 4-CMC (0.5 mM). Within a few min after 4-CMC addition, which remained in the medium, iron-treated neurons displayed a significantly higher fluorescence increase in the soma relative to the controls (Figure 1A). Following 4-CMC addition, fluorescence kept increasing since RyR channels remain open in the constant presence of 4-CMC, releasing Ca^2+^ and prompting as well Ca^2+^ entry from the extracellular solution via store-operated Ca^2^^+^ channels. These combined effects promote a sustained increase in calcium with time, which is much faster in cells pre-incubated with Fe-NTA. Fluorescence images taken from a control culture before 4-CMC addition (Figure 1B), and at the time of peak fluorescence induced by 4-CMC (Figure 1C), show that this RyR agonist increased fluorescence both in neuronal soma and neurites. Neurons from a culture pre-incubated with Fe-NTA had similar basal fluorescence as control neurons (Figure 1D), but displayed a much larger maximal fluorescence increase both in soma and neurites after 4-CMC addition (Figure 1E). On average, addition of 4-CMC increased 1.6-fold the maximal fluorescence recorded in the soma of control neurons and 3.2-fold in the soma of neurons treated with Fe-NTA (Figure 1F). These results indicate that prolonged exposure to Fe-NTA favored agonist-induced RyR-mediated Ca^2^^+^ release, presumably via RyR redox modifications produced by iron-generated ROS.

### IRON INDUCES MITOCHONDRIAL FISSION IN PRIMARY HIPPOCAMPAL NEURONS, DETERMINED BY MORPHO-TOPOLOGICAL ANALYSIS OF THEIR MITOCHONDRIAL NETWORK

**FIGURE 2:**
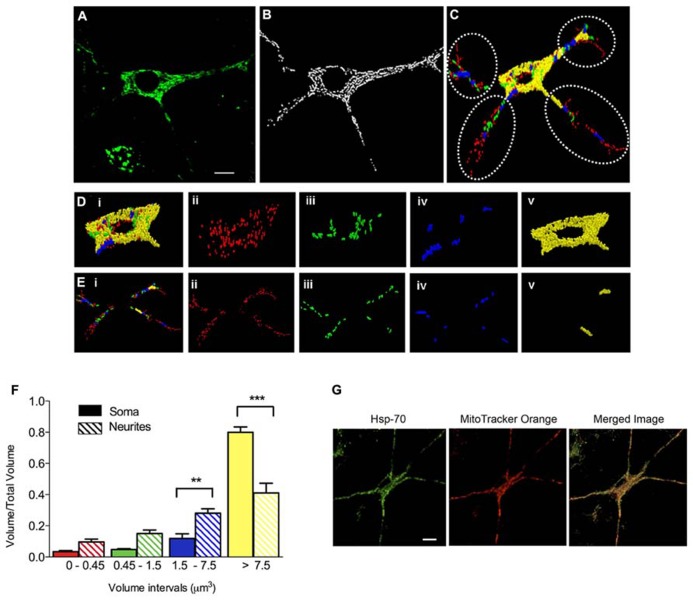
**Clustering of the mitochondrial network in primary hippocampal neurons**. **(A)** Representative image of Hsp-70 immuno-fluorescence (green), shown as a marker of the mitochondrial network in a control neuron. **(B)** Segmentation of mitochondria. **(C)** Clustering analysis by volume intervals after 3-D reconstruction. Colors represent volume intervals: red <0.45 μm^3^; green from 0.45 to 1.5 μm^3^; blue from 1.5 to 7.5 μm^3^, and yellow >7.5 μm^3^. Dotted circles separate neurites from soma. Images illus-trated in **(Di–v)** were taken in the soma. **(Di)** All mitochondrial clusters; **(Dii)** elements with volumes corresponding to 1–3 fused individual mitochondria (<0.45 μm^3^); **(Diii)** elements with 4–10 mitochondria (0.45–1.5 μm^3^); **(Div)** 11–50 mitochondria (1.5–7.5 μm^3^); and **(Dv)** >50 mitochondria (>7.5 μm^3^). The images illustrated in **(Ei–v)** were taken in neurites. **(Ei)** Mitochondrial clusters in neurites. **(Eii)** 1–3 mitochondria (<0.45 μm^3^), **(Eiii)** 4–10 mitochondria (0.45–1.5 μm^3^), **(Eiv)** 11–50 mitochondria (1.5–7.5 μm^3^), and **(Ev)** >50 mitochondria (>7.5 μm^3^). **(F)** Relative volume of mitochondrial clusters in soma (solid bars) and neurites (hatched bars). Values represent Mean ± SE (*n* = 3 independent cultures; in each culture, three cells were analyzed by condition). Statistical significance was analyzed by one-way ANOVA followed by Bonferroni’s *post hoc* test. **p < 0.005, ***p < 0.0005. **(G)** Representative images of Hsp-70 immunofluorescence (green) and MitoTracker Orange staining (red) in a control neuron. The scale bars in **(A,G)** represent 10 μm.

We evaluated next if prolonged exposure of cultured hippocampal neurons to iron affected the structure of the mitochondrial network, determined by immunofluorescence analysis of the mitochondrial protein Hsp-70. In control conditions, the mitochondrial network of primary hippocampal neurons was highly interconnected with elongated mitochondria that extended across the cell body and neuronal projections (Figure 2A). Immunofluorescence images were subjected to segmentation studies (Figure 2B) and subsequent 3D reconstruction followed by morpho-topological analysis of these images (Figure 2C). The morpho-topological analysis defined four mitochondrial clusters according to their volume. The mean volume of single mitochondria (0.15 ± 0.04 μm^3^) was used to define all clusters (see materials and methods and Figure 2C). The largest mitochondrial cluster, with volumes >7.5 μm^3^, was the most dominant group in cell bodies (Figure 2D, images i–v), while neuronal projections contained a larger number of the smaller mitochondrial clusters (Figure 2E, images i–v). Nevertheless, the largest mitochondrial cluster accumulates the largest proportion of the total mitochondrial volume within neuronal projections. Results from 3 independent cultures (Figure 2F) illustrate that mature hippocampal neurons (18–21 DIV) display a characteristic organization of the mitochondrial network that may reflect specific cellular demands (Knott et al., 2008). Staining mitochondria with MitoTracker Orange yielded the same labeling pattern as Hsp-70 (Figure 2G).

**FIGURE 3:**
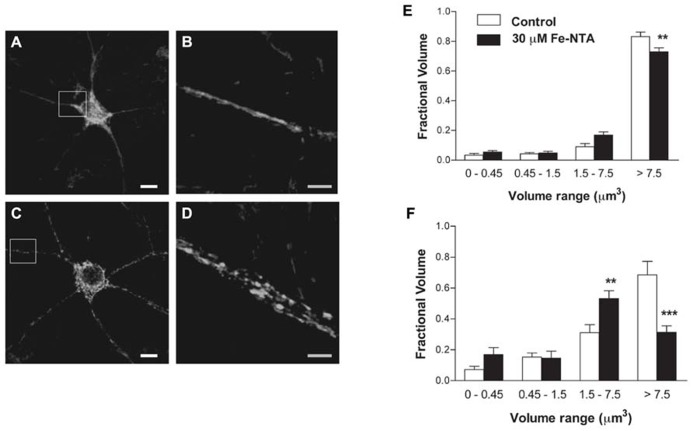
**Iron induces fragmentation of the mitochondrial network**. **(A)** Representative image of Hsp-70 immunofluorescence (green) used as a marker of the mitochondrial network in a control neuron. **(B)** Amplification of the white box in **(A)**. **(C)** Representative image of Hsp-70 immunofluorescence (green) in a neuron from a culture treated with 30 μM Fe-NTA for 24 h. **(D)** Amplification of the white box in **(C)**. Analysis of the mitochondrial clusters in soma **(E)** and neurites **(F)**: empty bars correspond to controls and black bars, after treatment with Fe-NTA. Calibration bars in **(A,C)** correspond to 10 μm; in **(B,D)**, to 5 μm. Values represent Mean ± SE (*n* = 3 independent cultures; ≥3 cells analyzed per condition). Statistical significance was analyzed by one-way ANOVA followed by Bonferroni’s *post hoc* test. **p < 0.005; ***p < 0.0001.

We investigated whether neurons treated with Fe-NTA for 24 h presented alterations in their mitochondrial network. Compared to a representative control neuron, which showed an interconnected mitochondrial network in soma and neurites (Figures 3A,B), a neuron incubated for 24 h with 30 μM Fe-NTA exhibited a significant loss in the continuity of their mitochondrial network, with an increased proportion of small mitochondria in soma and neurites (Figures 3C,D). As detailed below, a sizable fraction of neurons exhibited this behavior. Morpho-topological analysis of neurons with fragmented mitochondria from three independent cultures revealed that neurons in response to iron treatment exhibited a decreased fraction of the largest mitochondria cluster (>7.5 μm^3^) and an increased fraction of the intermediate cluster (1.5–7.5 μm^3^), both in the cell body and in the neuronal projections (Figures 3E,F).

**FIGURE 4:**
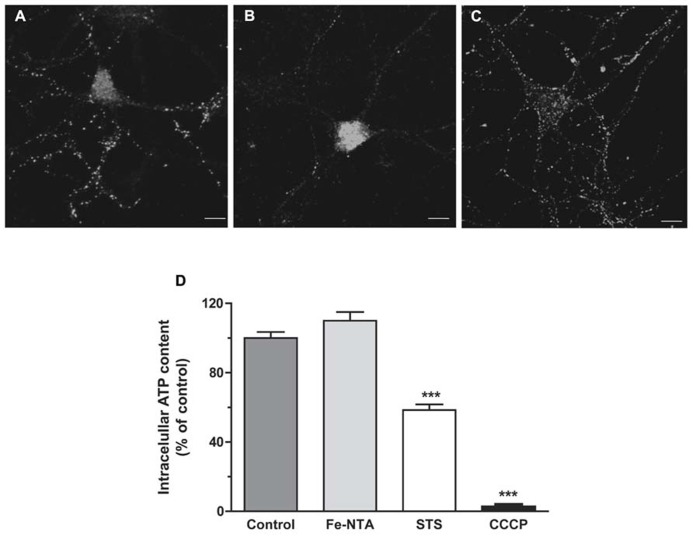
**Top panels: cytochrome c release**. **(A) **Representative images of cytochrome c immunofluorescence taken from a control neuron; **(B)** a neuron treated with 1 μM staurosporine (STS) for 5 h; **(C)** a neuron treated with 30 μM Fe-NTA for 24 h. **(D)** intracellular ATP content expressed relative to the control. Control cultures produced on average 0.65 ± 0.21 pmol ATP per well (containing 90,000 cells); this value was taken as 100%. Cultures were treated with 30 μM Fe-NTA for 24 h, with 1 μM staurosporine (STS) for 5 h, or with 10 μM CCCP for 30 min. Values represent Mean ± SE (*n* = 3 independent cultures, five wells analyzed per culture). Statistical significance was analyzed by one-way ANOVA followed by Bonferroni’s *post hoc* test. ***p < 0.0001.

As illustrated in Figures 4A–C, treatment with 30 μM Fe-NTA for 24 h did not induce cytochrome c release from the mitochondria to the cytoplasm, evaluated by immunofluorescence, whereas treatment with staurosporine produced massive cytochrome c release. Treatment with iron did not affect ATP production when compared to controls (Figure 4D). These combined results indicate that iron treatment did not induce apoptosis and did not alter significantly mitochondrial function.

### IRON-INDUCED MITOCHONDRIAL FISSION REQUIRES RyR-MEDIATED Ca^2**+**^ RELEASE

**FIGURE 5:**
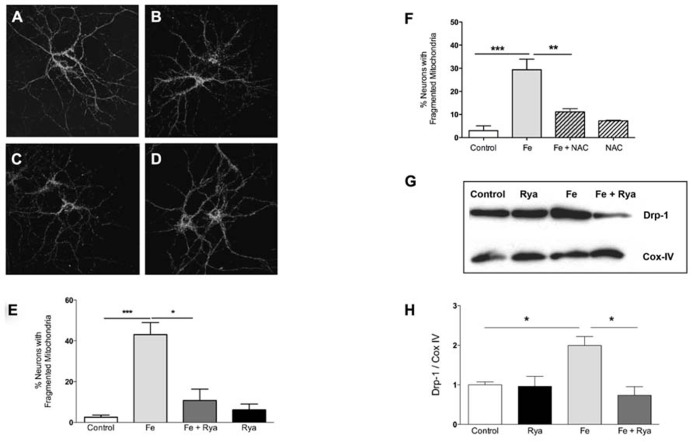
**Iron-induced fragmentation of the mitochondrial network requires RyR-mediated Ca^2^^+^ release**. Representative Hsp-70 immuno-fluorescence used as a marker of the neuronal mitochondrial network. **(A)** controls; **(B)** after treatment with 30 μM Fe-NTA; **(C)** after treat-ment with 30 μM Fe-NTA in the present of 50 μM ryanodine, and **(D)** treated with 50 μM ryanodine. Calibration bar = 20 μm. **(E)** Quantifi-cation of the percentage of neurons with fragmented mitochondria. **(F)** Addition to the culture medium of 10 mM NAC 30 min before treat-ment with Fe-NTA for 24 h protects against the mitochondrial fragmen-tation produced by Fe-NTA. **(G)** Representative Western blot and **(H)** quantification of Drp-1 and COX IV protein levels in sub-cellular fractions enriched in mitochondrial proteins; COX IV was used as loading control. Values in **(E)** represent Mean ± SE (*n* = 4 independent cultures, 35–40 cells analyzed per culture); values in **(F)** represent Mean ± SE (*n* = 3 independent cultures, 15 cells analyzed per culture Statistical signifi-cance was analyzed by one-way ANOVA followed by Bonferroni’s *post hoc* test. In **(H)**, the results from 3 independent Western blots (from 3 independent cultures) represent Mean ± SE. In this case, we analyzed statistical significance by repeated measured ANOVa followed by Newman–Keuls *post hoc* test. *p < 0.05; **p < 0.005; ***p < 0.0005.

As determined by immunofluorescence against the mitochondrial protein Hsp-70 (SanMartin et al., 2012), most control neurons presented a continuous mitochondrial network in soma and dendrites (Figure 5A). Treatment with Fe-NTA induced mitochondrial fission in neuronal cells (Figure 5B). Previous studies indicate that sustained increases in cytoplasmic [Ca^2^^+^] favor mitochondrial fission (Hom et al., 2007). Inhibition of RyR-mediated Ca^2^^+^ release with ryanodine prevents caffeine-induced responses in primary hippocampal neurons (Uneyama et al., 1993). To investigate if RyR-mediated Ca^2^^+^ release contributes to the increased mitochondrial fission induced by Fe-NTA, prior to treatment with Fe-NTA for 24 h RyR activity was inhibited by pre-incubating cultures for 4 h with 50 μM ryanodine (Uneyama et al., 1993; Adasme et al., 2011). Treatment with Fe-NTA was less effective in causing neuronal mitochondrial fission in cultures pre-incubated with ryanodine (Figure 5C), whereas cultures pre-incubated only with ryanodine displayed scarce mitochondrial fission (Figure 5D). Average results from 4 independent cultures indicate that only 3% of controls neurons exhibited fragmented mitochondria whereas treatment with Fe-NTA induced mitochondrial fission in 43% of the neuronal population (Figure 5E). In ryanodine treated cultures, on average 11% of neuronal cells incubated with Fe-NTA for 24 h displayed mitochondrial fission, while in cultures treated only with ryanodine 6% of neurons, on average, displayed fragmented mitochondria (Figure 5E).

In neuronal cells, RyR-mediated Ca^2^^+^ release is highly sensitive to cellular redox state and does not occur under reducing conditions (Hidalgo and Donoso, 2008). The general antioxidant agent NAC is a cellular precursor of glutathione. We have shown previously that pre-incubation of primary hippocampal neurons with NAC abolishes the stimulation of RyR-mediated Ca^2^^+^ release induced by 4-CMC (Paula-Lima et al., 2011); NAC also prevents the mitochondrial fragmentation induced by 4-CMC (SanMartin et al., 2012). These combined results strongly suggest that NAC, by changing the cellular redox balance toward more reducing conditions, prevents RyR-mediated Ca^2^^+^ release and the mitochondrial fragmentation caused by the ensuing intracellular [Ca^2^^+^] increase. Accordingly, we tested next the effects of NAC on iron-induced mitochondrial fragmentation. As illustrated in Figure 5F, NAC treatment reduced by 70%, on average, iron-induced mitochondrial fragmentation.

To evaluate the participation of the molecular machinery involved in the fission process in the change in mitochondrial structure induced by Fe-NTA, changes in the sub-cellular location of Drp-1 were evaluated by Western blot analysis of fractions enriched in mitochondrial proteins. Control mitochondrial fractions had a low basal level of Drp-1 in mitochondrial fractions, while cultures incubated with 30 μM Fe-NTA for 24 h displayed a significant increase in the Drp-1 mitochondrial content relative to the levels of the mitochondrial marker protein Cox-IV (Figures 5G,H). These results indicate that treatment with Fe-NTA induced the translocation of Drp-1 from the cytoplasm to the mitochondria. Cultures pre-incubated with 50 μM ryanodine prior to treatment with Fe-NTA for 24 h displayed significantly decreased mitochondrial Drp-1 levels, while ryanodine by itself did not affect the distribution of Drp-1 (Figures 5G,H).

Taken together, these results indicate that treatment with Fe-NTA promoted mitochondrial fission by inducing the translocation of Drp-1 to the mitochondria, via ROS-dependent RyR-mediated Ca^2^^+^ release from the ER.

### MITOCHONDRIA TAKE UP Ca^**2+**^ RELEASED VIA RyR CHANNELS

Mitochondria play a significant role in maintaining Ca^2^^+^ homeostasis, acting as an important intracellular Ca^2^^+^ buffer. In turn, mitochondrial Ca^2^^+^ signals control fundamental cellular functions, including energy metabolism and apoptotic cell death (Pacher et al., 2002). Changes in network structure may affect mitochondrial localization, modifying mitochondrial connections with the ER, mitochondrial Ca^2^^+^ uptake and intracellular Ca^2^^+^ homeostasis.

**FIGURE 6:**
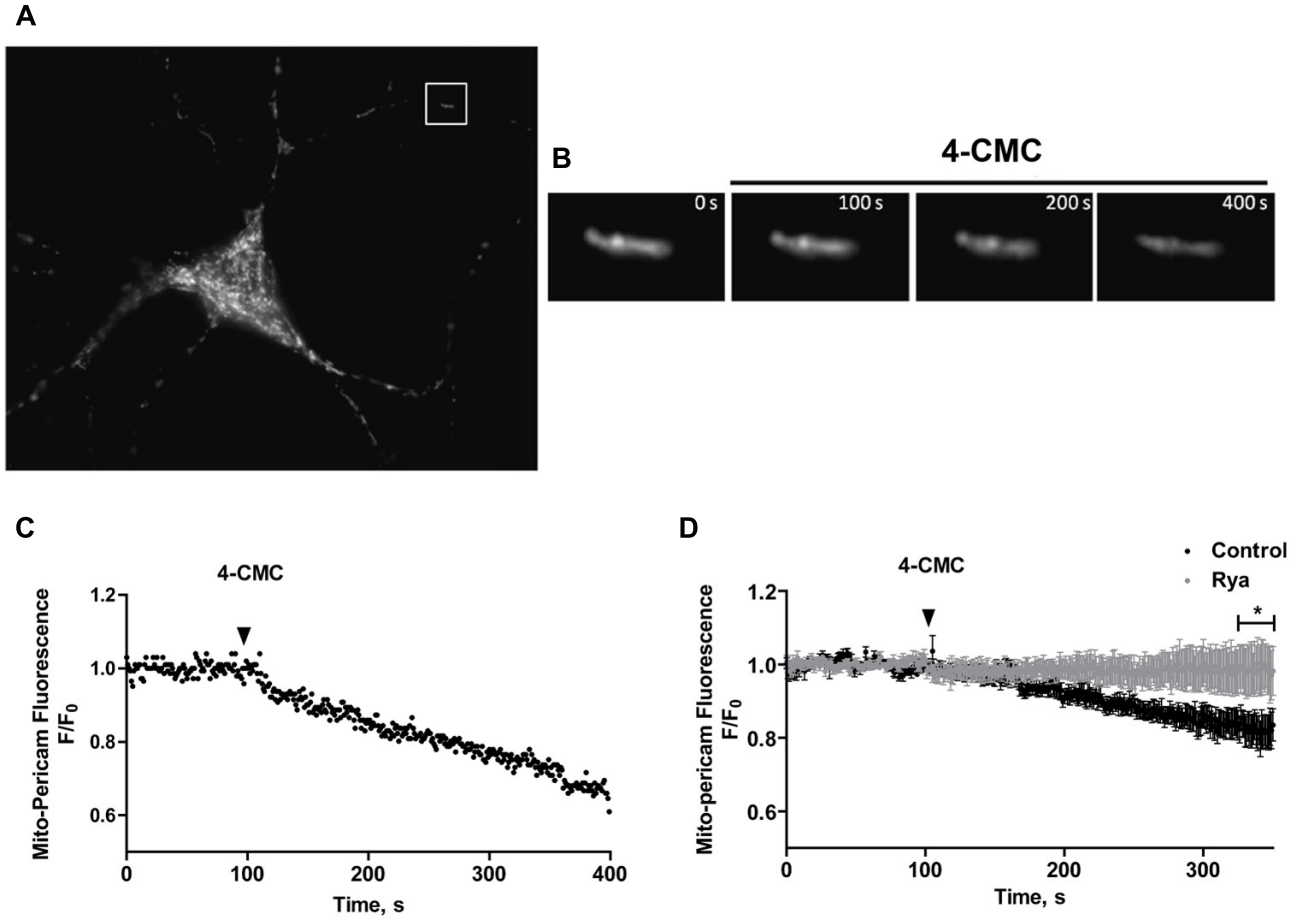
**Mitochondrial Ca^2^^+^ levels increase in response to RyR-mediated Ca^2+^ release**. **(A)** Representative fluorescence image of a hippocampal neuron expressing the mitochondrial Ca^2^^+^ sensor mito-pericam. **(B)** Amplification of the neuronal segment defined by the inset highlighted in **(A)**, taken at various times after addition of 0.5 mM 4-CMC. Calibration bars in **(A,B)** correspond to 10 μm and 0.5 μm respectively. **(C)** Representative time course of mito-pericam fluorescence changes in response to addition of 0.5 mM 4-CMC to a control neuron. **(D)** Time course of the average mito-pericam fluorescence changes induced by addition of 0.5 mM 4-CMC to control neurons (black symbols) or to neurons pre-incubated for 1 h with 50 μM ryanodine (gray symbols). Values represent Mean ± SE (*n* = 3 independent cultures, 4–5 cells analyzed per culture). Statistical significance was analyzed by two-way ANOVA followed by Bonferroni’s *post hoc* test. *p < 0.01.

To investigate if mitochondria take up Ca^2^^+^ released through RyR channels, we transfected neurons with mito-pericam, a plasmid expressing a protein sensitive to Ca^2^^+^ conjugated to a mitochondrial destination signal and a GFP derivative. Mitochondrial Ca^2^^+^ uptake decreases mito-pericam fluorescence due to a chromophore structural change (Rizzuto et al., 1992). Hippocampal neurons transfected with mito-pericam (Figure 6A) were stimulated with the RyR agonist 4-CMC to induce RyR-mediated Ca^2^^+^ release; fluorescence changes were recorded in a neuronal projection (inset, Figure 6A). Addition of 4-CMC produced a significant decrease in mito-pericam fluorescence (Figure 6B), which decayed linearly in the time recorded (Figure 6C). The results of this experiment show that RyR-mediated Ca^2^^+^ release effectively promoted mitochondrial Ca^2^^+^ uptake (Figures 6A–C). Average changes in mito-pericam fluorescence (n = 3) following 4-CMC addition are illustrated in Figure 6D (black circles).

To corroborate that RyR-mediated Ca^2^^+^ release stimulated by 4-CMC promoted mitochondrial Ca^2^^+^ uptake, cultures were pre-incubated for 1 h with inhibitory concentrations of ryanodine (50 μM) to prevent RyR-mediated Ca^2^^+^ release. Neurons in cultures pre-incubated with inhibitory ryanodine did not exhibit changes in mito-pericam fluorescence following 4-CMC addition (Figure 6D, gray circles). These results confirm that agonist-stimulated RyR channels generate the Ca^2^^+^ signals that induce mitochondrial Ca^2^^+^ uptake in primary hippocampal neurons, which as described in other studies (Csordas and Hajnoczky, 2009) requires proximity between these two organelles.

### MITOCHONDRIAL UPTAKE OF Ca^**2+**^ RELEASED BY RyR DOES NOT OCCUR IN MITOCHONDRIA FRAGMENTED BY IRON TREATMENT

**FIGURE 7:**
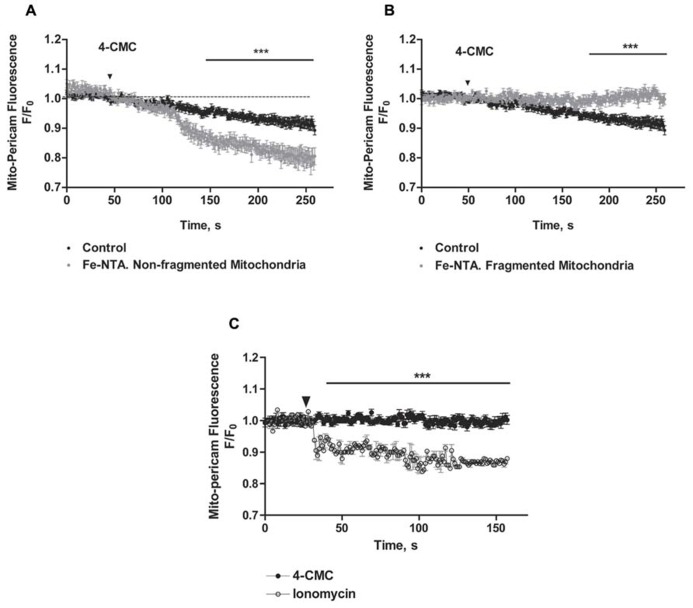
**The uptake of calcium released by RyR does not occur in fragmented mitochondria, which take up Ca^2+^ after ionomycin treatment**. Hippocampal neurons were transfected with mito-pericam. **(A)** Time course of changes in mito-pericam fluorescence before and after 4-CMC addition to control neurons (black symbols), or to neurons that displayed non-fragmented mitochondria after treatment for 24 h with 30 mM Fe-NTA (gray symbols). **(B)** Time course of changes in mito-pericam fluorescence before and after addition of 0.5 mM 4-CMC to control neurons (black sym-bols), or to neurons that displayed fragmented mitochondria after treatment for 24 h with 30 mM Fe-NTA (gray symbols). **(C)** Mito-pericam fluorescence changes recorded before and after addition at the arrow of 0.5 mM 4-CMC (black symbols) or of 100 mg/ml ionomycin (open symbols) to neurons with fragmented mitochondria after 24 h treatment with 30 mM Fe-NTA. Calibration bars correspond to 10 ìm. Values represent Mean ± SE (*n* = 3 independent cultures, 4–5 cells analyzed per culture). Statistical significance was analyzed by two-way ANOVA followed by Bonferroni’s *post hoc* test. ***p < 0.01.

We evaluated next if changes in mitochondrial network structure affected mitochondrial Ca^2^^+^ uptake induced by RyR-mediated Ca^2^^+^ release. We confirmed that mitochondria of control neurons, which have an elongated and interconnected structure, exhibit a decrease in mito-pericam fluorescence after addition of 4-CMC, indicating uptake by mitochondria of Ca^2^^+^ released from the ER via RyR channels (Figure 7A). Addition of 4-CMC to neurons that retained an intact mitochondrial network after Fe-NTA treatment caused a significantly higher mito-pericam fluorescence decrease with time (Figure 7A), indicating higher Ca^2^^+^ uptake rates. These findings agree with the larger RyR-mediated Ca^2^^+^ signals produced by 4-CMC in neurons treated with Fe-NTA (Figure 1F). In contrast, 4-CMC addition to neurons displaying fragmented mitochondrial networks after Fe-NTA treatment did not produce a change in mito-pericam fluorescence (Figure 7B). Yet, these mitochondria readily incorporated Ca^2^^+^ that enters the cell from the extracellular medium after addition of the Ca^2^^+^ ionophore ionomycin (Figure 7C). Jointly, these results suggest that RyR-mediated Ca^2^^+^ signals stimulate mitochondrial Ca^2^^+^ uptake and that this process requires an intact mitochondrial network.

## DISCUSSION

The number, distribution and morphology of mitochondria are characteristic of each cellular type and have a direct relationship with the function and energetic demands of the cell (Kuznetsov et al., 2009). In this context, due to the polarized structure of neurons the physical and temporal distribution of mitochondria has to meet the high-energy demands of ATP-dependent processes in soma, axon and neurites (Knott et al., 2008). The present morpho-topological analysis of hippocampal neurons in culture revealed different mitochondrial network structures in soma and neurites of control neurons. In the soma, mitochondria form a highly connected network characterized by large elongated structures. In neurites, however, we observed different structures and sizes of mitochondria, with a predominant mitochondrial population with volumes <7.5 μm^3^. These results indicate that the mitochondrial network is more heterogeneous and discontinuous in neurites, which contain more homogeneously distributed and smaller mitochondria. The presence of mitochondria with lower volumes in neurites than in the soma adds to previous studies showing that a fraction of small mitochondria moves to the dendritic protrusions and spines after electrical stimulation to promote synaptic plasticity (Li et al., 2004), highlighting the relationship between mitochondrial distribution, size, location and function in neurons.

### IRON-INDUCED MITOCHONDRIAL FRAGMENTATION REQUIRES RyR-MEDIATED Ca^2^^**+**^ RELEASE

The current results indicate that prolonged exposure (24 h of cultured hippocampal neurons) to iron promoted mitochondrial fission in soma and neurites in a significant fraction (43%) of the total neuronal population. Increasing cytoplasmic [Ca^2^^+^], either by increasing extracellular [Ca^2^^+^] in cells derived from the liver or the cardiovascular system (Yu et al., 2011) or by emptying the ER with thapsigargin in a rat liver cell line (Hom et al., 2007), promotes mitochondrial fission. We have reported previously that stimulation of RyR-mediated Ca^2^^+^ release with 4-CMC (SanMartin et al., 2012) or with the synaptotoxic Aβ oligomers (Paula-Lima et al., 2011) promotes mitochondrial fission in primary hippocampal neurons. Here, we show that RyR channels generate the Ca^2^^+^ signals underlying iron-induced mitochondrial fission, since inhibitory ryanodine concentrations decreased iron-induced mitochondrial fission and suppressed Drp-1 translocation to the mitochondria. These combined results confirm RyR involvement in neuronal mitochondrial dynamics and add to the previously reported roles of RyR channels in several central neuronal functions (Adasme et al., 2011; Paula Lima et al., 2014).

Translocation of the Drp-1 protein from the cytoplasm to the mitochondria is a requisite step of mitochondrial fission. Several post-translational modifications, which include Drp-1 phosphorylation (Cribbs and Strack, 2007), dephosphorylation (Cereghetti et al., 2008), ubiquitination (Karbowski et al., 2007), sumoylation (Harder et al., 2004) and S-nitrosylation (Cho et al., 2009), promote the translocation of Drp-1 to the mitochondria. We observed that prolonged exposure (24 h) of primary hippocampal cultures to iron-induced Drp-1 translocation to the mitochondria, which presumably caused the observed increase in mitochondrial fission displayed by neuronal soma and neurites. Iron promotes ROS production via the Haber–Weiss/Fenton reactions, which could contribute to trigger mitochondrial fission. In fact, other reports indicate that ROS stimulate mitochondrial fragmentation (Jahani-Asl et al., 2007; Yu et al., 2011). Treatment of primary hippocampal neurons with the antioxidant agent NAC prevents the mitochondrial fragmentation induced by the RyR agonist 4-CMC (Paula-Lima et al., 2011) or by iron (Figure 5F), indicating that mitochondrial fragmentation requires ROS. Yet, information on direct ROS-dependent Drp-1 modifications is not available, raising the possibility that iron-mediated ROS generation modifies other proteins, which might affect neuronal mitochondrial dynamics. The present results indicate that iron-induced Drp-1 translocation to the mitochondria requires ROS-responsive RyR-mediated Ca^2^^+^ release. Previous reports indicate that Ca^2^^+^ signals promote Drp-1 dephosphorylation by calcineurin (Cereghetti et al., 2008; Han et al., 2008), enhancing Drp-1 translocation to the mitochondria and mitochondrial fission. We propose that the increase in intracellular [Ca^2^^+^], resulting from the enhanced RyR-mediated Ca^2^^+^ release produced by iron-induced ROS generation, promotes calcineurin activation leading to Drp-1 dephosphorylation and translocation to the mitochondria. Albeit superoxide anion inhibits calcineurin activity (Rusnak and Mertz, 2000), in our conditions generation of superoxide anion by iron is thermodynamically unfavorable.

RyR channels possess cysteine residues that are highly reactive under physiological conditions, a property that makes them likely to act as intracellular redox sensors (Hidalgo et al., 2005). Controlled RyR oxidation stimulates RyR activity in hippocampal (Kemmerling et al., 2007) and cortical neurons (Bull et al., 2007, 2008), and underlies RyR-mediated Ca^2^^+^-induced Ca^2^^+^ release from the ER in electrically stimulated hippocampal neurons (Riquelme et al., 2011). We have reported previously that iron, by promoting ROS generation, stimulates RyR-mediated Ca^2^^+^ release in primary hippocampal neurons (Munoz et al., 2011), leading to the activation of signaling pathways required for synaptic plasticity. Consequently, we propose that the intracellular [Ca^2^^+^] increase due to RyR stimulation via iron-generated ROS causes the augmented mitochondrial fission displayed by iron-treated neurons. Our observation that increasing the iron load causes mitochondria fission connects with previous observations showing that treatment of Chang cells with the highly selective iron chelator desferrioxamine results in significantly elongated mitochondria (Yoon et al., 2006).

It is important to note that the changes in mitochondrial network structure induced by iron did not affect ATP production nor induced apoptosis. These results show that in our experimental model, iron did not promote mitochondrial damage since fragmented mitochondria remained metabolically active. Mitochondrial fission induced by iron may increase the number of mitochondria available for axonal transport or toward spots of high-energetic demand like the synapse, close to functional Ca^2^^+^ stores that provide Ca^2^^+^ for activation of oxidative metabolism (Kann et al., 2003). We cannot exclude, however, that longer times of incubation with iron might produce deleterious effects on mitochondrial function.

### STIMULATION OF RyR-MEDIATED Ca^2**+**^ RELEASE PROMOTES Ca^2**+**^ UPTAKE ONLY IN NON-FRAGMENTED MITOCHONDRIA

Previous reports indicate that RyR stimulation promotes the emergence of rapid and simultaneous Ca^2^^+^ signals in cytoplasm and mitochondria in cardiac muscle cells (Szalai et al., 2000; Garcia-Perez et al., 2008). Our results show that RyR activation by the agonist 4-CMC promoted fast mitochondrial Ca^2^^+^ uptake in control neurons bearing elongated and interconnected mitochondria. Moreover, compared to control neurons, neurons that possessed non-fragmented mitochondria after iron treatment displayed enhanced mitochondrial Ca^2^^+^ uptake in response to the RyR agonist 4-CMC. This finding may reflect an increased oxidative tone in iron-treated neurons, which would facilitate the redox-sensitive Ca^2^^+^ release induced by the RyR agonist 4-CMC (Paula-Lima et al., 2011). In contrast, RyR activation by 4-CMC did not promote mitochondrial Ca^2^^+^ uptake in neurons exhibiting fragmented mitochondria after iron treatment, albeit the Ca^2^^+^ uniporter activity of mitochondria fragmented by iron remained functional since the global increase in cytoplasmic Ca^2^^+^ caused by the Ca^2^^+^ ionophore ionomycin prompted fast mitochondrial Ca^2^^+^ uptake. Skeletal myotubes also display less effective mitochondrial Ca^2^^+^ uptake after fission; electrical stimulation of myotubes promotes the emergence of RyR-dependent mitochondrial Ca^2^^+^ signals, and over-expression of Drp-1 induces mitochondrial fission and decreases mitochondrial Ca^2^^+^ uptake in response stimulation (Eisner et al., 2010). These previous results indicate that RyR-mediated mitochondrial transfer of Ca^2^^+^ is relevant for the control of oxidative metabolism and likely allows local feedback regulation of cytoplasmic Ca^2^^+^ signaling through mitochondria, both in cardiac and skeletal muscle (Eisner et al., 2013). All combined, these results support the hypothesis that mitochondrial fission directly affects the interaction between ER and mitochondria, decreasing the transmission of Ca^2^^+^ signals generated by RyR-mediated Ca^2^^+^ release to the mitochondria.

We propose that fragmented mitochondria loose their physical interactions with the ER, resulting in decreased functional coupling between RyR channels and the mitochondrial Ca^2^^+^ uniporter since if both organelles are not close enough the generation of Ca^2^^+^ microdomains caused by RyR-mediated Ca^2^^+^ release might not be sufficient to activate the mitochondrial Ca^2^^+^ uniporter. Consequently, fragmented mitochondria are likely to lose their intracellular Ca^2^^+^ buffering function in response to RyR-mediated Ca^2^^+^ signals. This issue might have important consequences for intracellular Ca^2^^+^ homeostasis in neurons since an imbalance toward mitochondrial fission, which occurs in neurodegenerative diseases (Itoh et al., 2013), would promote the loss of the Ca^2^^+^ buffering function of mitochondria resulting in increased cytoplasmic Ca^2^^+^signals. In fact, neurons from transgenic rodents that develop Alzheimer’s disease (3xTg-AD) exhibit increased intracellular resting [Ca^2^^+^] (Lopez et al., 2008), which may arise at least in part from increased mitochondrial fragmentation.

In conclusion, we found that iron treatment promoted mitochondrial fission through a process that required functional RyR channels, and that fragmented mitochondria did not take up Ca^2^^+^ in response to RyR-mediated Ca^2^^+^ release. The loss of mitochondrial Ca^2^^+^ uptake exhibited by fragmented mitochondria, which is likely to disrupt cellular Ca^2^^+^ homeostasis, presumably contributes to the increased fragility observed in neurons subjected to a high iron oxidative load, as observed in Parkinson’s and Alzheimer’s disease. The present results add to previous reports showing significant contributions of RyR-mediated Ca^2^^+^ release from the ER to neuronal function, including synaptic plasticity (Bardo et al., 2006) and hippocampal-dependent spatial memory processes, as discussed at length in two recent comprehensive reviews (Baker et al., 2013; Paula Lima et al., 2014).

## Conflict of Interest Statement

The authors declare that the research was conducted in the absence of any commercial or financial relationships that could be construed as a potential conflict of interest.
